# Hemophagocytic Lymphohistiocytosis and Hodgkin Lymphoma in a Newly Diagnosed HIV Patient: A Diagnostic Dilemma

**DOI:** 10.7759/cureus.41127

**Published:** 2023-06-29

**Authors:** Tobechukwu J Okobi, Sandhya Cautha, Tanushree Bhatt, Irhoboudu Dickson Atogwe, Patrik Schmidt, Dhruv Patel, Misbahuddin Khaja

**Affiliations:** 1 Internal Medicine, Bronx Care Health System, Bronx, USA; 2 Internal Medicine/Pulmonary Critical Care, Icahn School of Medicine at Mount Sinai/Bronx Care Health System, Bronx, USA

**Keywords:** malignancy, hodgkin lymphoma, ebv infection, hiv, hemophagocytic lymphohistiocytosis

## Abstract

Hemophagocytic lymphohistiocytosis (HLH) is a severe hyperinflammatory syndrome that arises due to a dysregulated immune response caused by the aberrant activation of lymphocytes and macrophages. In this rare case report, we describe a newly diagnosed human immunodeficiency virus (HIV) patient who was found to have Epstein-Barr virus (EBV) provoked HLH and co-existing Hodgkin lymphoma (HL).

Our patient was a 28-year-old newly diagnosed HIV patient who presented with nonspecific symptoms, including bilateral foot pain and tingling sensation. Laboratory findings were significant for pancytopenia. With a high index of suspicion, the patient had a bone marrow biopsy done which confirmed a diagnosis of both HLH and Hodgkin's lymphoma. The case highlighted the diagnostic dilemma of HLH in the setting of HIV infection. Identifying the major components of his disease process was pivotal to ensure that the patient was commenced on appropriate therapy for the EBV-driven HLH and HL.

The diagnosis of HLH in newly diagnosed HIV remains challenging due to the diverse clinical presentations and the need to exclude other possible causes. The clinical features of HLH, HL, and HIV can be nonspecific and overlap, creating a diagnostic dilemma. Diagnosis requires a combination of clinical, laboratory, and histopathological features. The management in such cases requires prompt diagnosis through a multidisciplinary approach, a variety of chemotherapy, immunosuppression, supportive care, and treatment of the underlying triggers.

## Introduction

Hemophagocytic lymphohistiocytosis (HLH) is a rare and life-threatening disease associated with severe hyperinflammatory syndrome which can be either primary or secondary [1]. It was first described in the medical literature in the 1950s, but it wasn't until the 1980s that consistent diagnostic criteria and treatment approaches were established. The disease arises due to a dysregulated immune response caused by the aberrant activation of lymphocytes and macrophages [2]. It can affect people of all ages and can be triggered by various underlying diseases or infections. Its incidence and prevalence within the population are highly variable due to multiple confounders that can impact the identification and diagnosis of the disease [2]. Also, mortality varies depending on the underlying cause, the severity of the disease, and the availability of specialized care [2]. Diagnosing HLH can be very challenging, especially in adults, due to its rarity, complexity, and nonspecific clinical features requiring a high suspicion index. Early recognition, prompt initiation of therapy, and close monitoring of complications are essential to improve the outcomes of HLH. In this report, we highlight a case of a newly diagnosed human immunodeficiency virus (HIV) patient who presented with non-specific symptoms and labs concerning pancytopenia. He was worked up and found to have Epstein-Barr virus (EBV) provoked HLH and Hodgkin lymphoma (HL). With this case, we aim to provide an overview of HLH, including epidemiology, pathogenesis, clinical features, diagnosis, and treatment, while identifying the challenges surrounding the diagnosis and management of HLH.

## Case presentation

The patient was a 28-year-old male with a history of sickle cell trait, newly diagnosed HIV, and active hepatitis B who presented with worsening bilateral lower extremity numbness, cough, shortness of breath, and diarrhea for five days. The patient had no significant family history. He was recently commenced on elvitegravir, cobicistat, emtricitabine, and tenofovir. Upon arrival, he had mild respiratory distress requiring oxygen via nasal cannula. Other physical examinations were normal. Laboratory tests showed severe pancytopenia and lactic acidosis (Table 1), HIV RNA of 13,600 copies/ml, and an absolute CD4 count of <20. Peripheral blood smear showed anisopoikilocytosis but no schistocytes. Chest x-ray showed a left lower lobe consolidation.

**Table 1:** Relevant Laboratory Results WBC: white blood cell, INR: international normalized ratio.

Laboratory Parameters	Results	Reference/SI Unit
Hematology		
Hemoglobin	4.3	12.0-16.0g/dl
Platelet	38	150-400k/ul
WBC	2.0	4.8-10.8k/ul
Neutrophil Count	1.4	1.5-8.0k/ul
Lymphocyte Count	0.3	1.0-4.8k/ul
Chemistry		
Triglyceride	301	46-150mg/dL
Ferritin	47563	13.0-150.0 ng/mL
Coagulation Profile		
Prothrombin Time	22.4	9.9-13.3 seconds
INR	1.87	.85-1.14
Fibrinogen	242	185.0-450.0mg/dL

**Figure 1:**
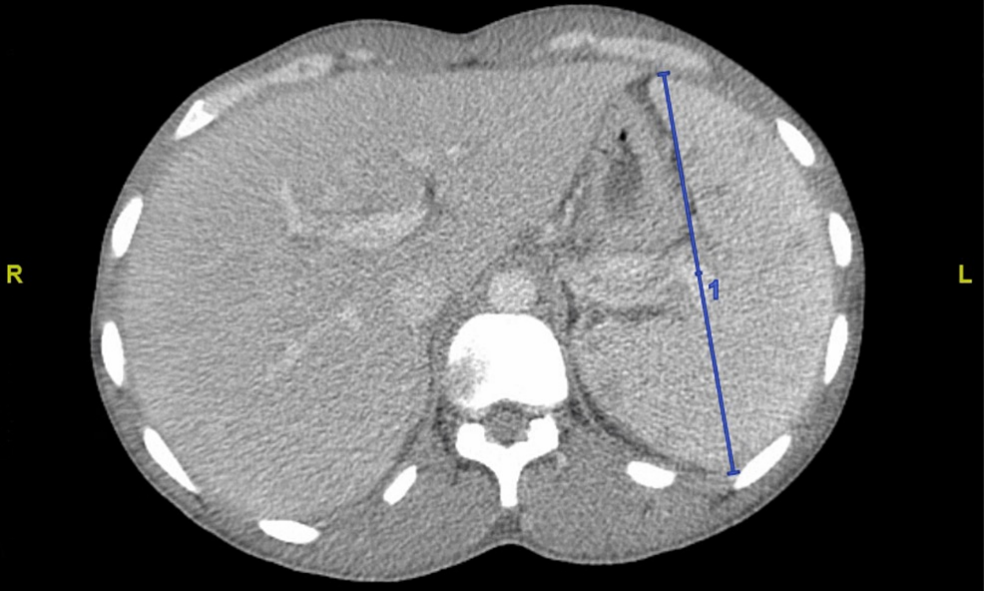
Computed Tomography of the Abdomen Showing Splenomegaly

A computed tomography (CT) of the abdomen showed splenomegaly (Figure 1) and enlarged retroperitoneal lymph nodes. The CT head did not show any abnormality. Sputum culture grew methicillin-sensitive staphylococcus aureus. He was commenced on broad-spectrum antibiotics and pneumocystis jiroveci virus prophylaxis. Antiretrovirals were held due to concerns for immune reconstitution inflammatory syndrome. During his hospital course, his blood counts declined, with worsening anemia, thrombocytopenia, and coagulopathy requiring multiple transfusions. He received four doses of dexamethasone and two doses of intravenous immune globulin for suspected HIV-associated thrombocytopenia with no significant improvement. He continued to have numerous fever spikes with hypotension and worsening mentation requiring initiation of pressors and intubation. He also developed worsening renal function requiring dialysis. 

**Figure 2:**
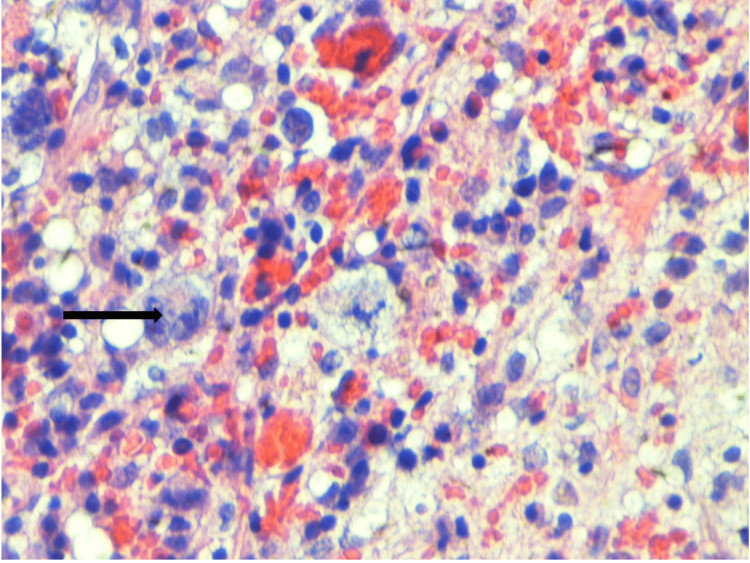
The Marrow Smear Shows Numerous Histiocytes Many of Them Showing Evidence of Phagocytosis of Erythrocytes

**Figure 3:**
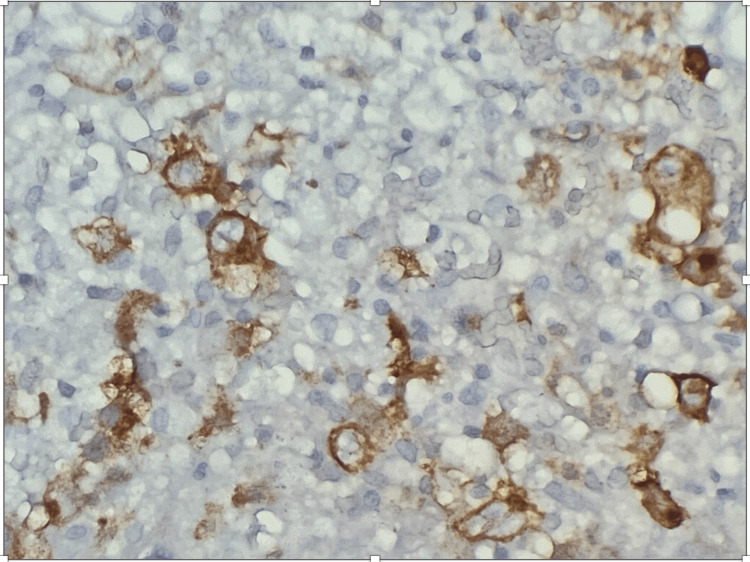
CD30 Positive Hematolymphoid Large Cells

**Figure 4:**
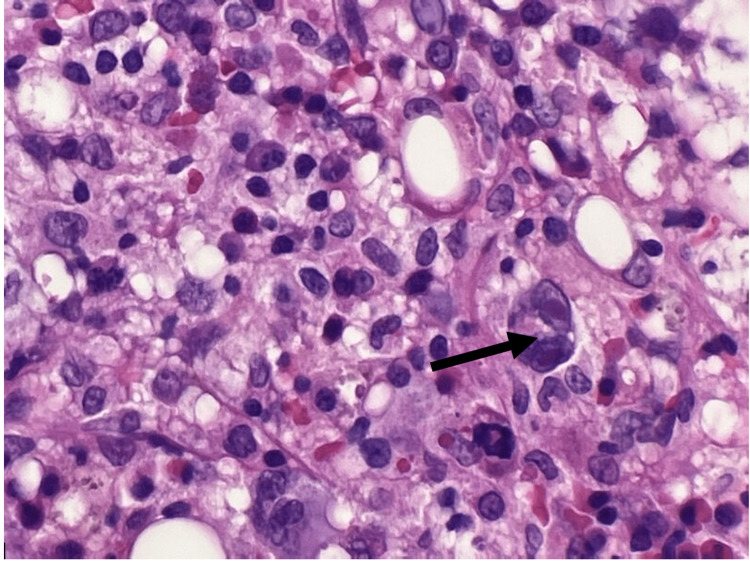
Reed-Sternberg Cell on Hematoxylin and Eosin Stain

Due to deteriorating pancytopenia, he had a bone marrow biopsy which showed EBV+ infarction necrosis with hemophagocytic activity and diminished trilineage hematopoiesis suggestive of HLH (Figure 2). Further analysis showed bone marrow involvement by CD30-positive hematolymphoid cells suggestive of Hodgkin's lymphoma (Figures 3, 4). EBV ​polymerase chain reaction (PCR) from bone marrow showed 1,054,516 copies per milliliter. As EBV was thought to induce phagocytosis of the blood cells, he was commenced on acyclovir. Also, rituximab was started to eradicate the CD20 cells serving as the EBV reservoir, and tenofovir was continued for HBV treatment. The patient responded well to treatment, with increased blood counts and a dramatic decline in EBV DNA levels. He was extubated and transferred to another facility for further diagnostic workup and therapy, including genetic testing for hereditary HLH and a possible bone marrow transplant. 

## Discussion

HLH is a rare and life-threatening syndrome characterized by the activation of cytotoxic T cells and macrophages, resulting in hypercytokinemia [3] and pathologic inflammation involving various organ systems. The disease is sporadic, with an incidence of about 1-2 cases per million in adults [4, 5] and a survival rate of <10% in the pre-immuno-chemotherapeutic era [6]. Primary HLH is more common in children and is usually caused by genetic mutations that affect immune regulation and lymphocyte cytotoxicity. In contrast, secondary HLH is more common in adults and is generally triggered by infections such as EBV, malignancies (most commonly lymphoma [7]), and immunodeficiency (HIV) [1, 8-9]. Pathogenesis in both forms is similar, with a dysregulated immune response resulting in uncontrolled cytokine release and systemic inflammation. 

The association between HLH and HIV infection has been described in a few reports [10-11]. These reports suggest that HLH can be a rare but severe complication of acute HIV infection resulting in increased mortality, particularly in individuals with pre-existing conditions or receiving immunosuppressive therapy [1]. One case report described a 62-year-old male with Henoch-Schönlein purpura under immunosuppressive treatment who presented with HLH symptoms two weeks after the onset of acute HIV infection [1]. Another case report described a 45-year-old male with life-threatening HLH due to an acute HIV infection [10]. Diagnosing HLH in the setting of HIV can be challenging due to the overlap in the presentation of HLH and advanced HIV infection [12]. HLH is more frequent in HIV-associated lymphomas compared to non-HIV-associated lymphomas. Patients with HL, lymphoma with bone marrow infiltration, and HHV-8-associated lymphoma have a higher likelihood of developing HLH with remarkably higher mortality with HLH present [13]. 

EBV reactivation is common in HIV-infected individuals due to their weakened immune systems [14]. It plays a crucial role in the pathogenesis of HLH [7, 15]^ ^and HL. EBV-associated HLH is one of the severe effects of EBV reactivation in immunocompromised patients [16], with exceptionally high fatality rates [17]. In these patients, EBV is frequently detected in the bone marrow, liver, and spleen, indicating its role in the pathogenesis of the disease [14]. Identifying the role of EBV in HLH as well as HL have both management and prognostic implications, as the early introduction of anti-EBV agents could change the clinical course of the disease. 

Clinical features of HLH 

HLH can affect individuals of all ages, but the primary form is more common in children [1]. The clinical features are nonspecific and can resemble other hyperinflammatory syndromes like sepsis [18]. Patients with HLH may present with fever, cytopenias, hepatosplenomegaly, lymphadenopathy, and coagulation abnormalities [1]. Neurological symptoms like seizures and altered sensorium may also be seen in severe cases. 

Diagnosis 

The diagnostic criteria for HLH have continued to evolve with various nomenclatures developed over the years. The current diagnostic criteria in adults are derived mainly from the Histiocyte Society diagnostic criteria initially described in 1991 and updated in 2004 [1] The criteria include clinical, laboratory, and histopathological features. The diagnosis of HLH requires five out of nine criteria, including fever, splenomegaly, cytopenias, hypertriglyceridemia, hyperferritinemia, hemophagocytosis in bone marrow, spleen, or lymph nodes, low or absent natural killer cell activity, and elevated soluble CD25 levels [1]. Soluble CD25 [sCD25] (also known as Soluble interleukin-2 (IL-2) receptor) has recently been reported as a good to an excellent low-cost diagnostic test for adult HLH, with an area under the curve of 0.90 (95% confidence interval, 0.81-0.99) [1]. Further studies are needed to evaluate the utility of sCD25 as a diagnostic tool in HIV-associated HLH. These criteria, however, are not specific to HLH and can be observed in other diseases, posing a significant challenge in managing HLH [1]. Furthermore, the diagnosis can be challenging without a known trigger, and the diagnostic criteria may need to be modified in such cases [18]. 

Diagnostic challenges in HIV-associated HLH 

While diagnosing HLH is already challenging, it can be even more complex in HIV-associated HLH. One of the challenges in diagnosing HIV-associated HLH is the lack of specific diagnostic criteria for the disease. As HLH is a rare disease, there are no widely accepted diagnostic criteria for HIV-associated HLH, and the diagnosis is often based on clinical suspicion and the exclusion of other possible causes of fever, cytopenias, and organ dysfunction [19]. Another challenge in diagnosing HIV-associated HLH is the overlapping symptoms with other HIV-related conditions. Patients with HIV infection can develop a wide range of opportunistic infections and malignancies, presenting symptoms similar to HLH, such as fever, cytopenias, hepatosplenomegaly, and lymphadenopathy. Therefore, a thorough differential diagnosis is crucial to exclude other possible causes of these symptoms, and diagnostic tests, including laboratory investigations and imaging studies, are often required to confirm the diagnosis of HIV-associated HLH [9]. A multidisciplinary approach involving infectious diseases, hematology, immunology, and pathology experts is necessary for prompt management. 

Management 

The management of HLH involves a combination of chemotherapy, immunosuppression, and supportive care [1]. Therapy aims to suppress the immune response, control the underlying trigger, and prevent relapse. Treating with cytotoxic agents like etoposide in combination with dexamethasone is required in severe cases. Supportive care includes measures to manage complications like disseminated intravascular coagulation and multi-organ dysfunction [1, 2]. 

Case analysis 

Our case highlighted the diagnostic dilemma of HLH in the setting of HIV infection. His presentation was non-specific in the setting of newly diagnosed HIV. With worsening pancytopenia and a high index of suspicion, the patient was worked up for HLH. His biopsy result and EBV PCR DNA confirmed a diagnosis of EBV-driven HLH with HL. Identifying the major components of his disease process was pivotal to ensure that the patient was commenced on appropriate anti-viral medication for the EBV while making adequate arrangements to begin immuno-chemotherapy for his HLH. 

## Conclusions

The initial treatment of HLH includes controlling the cytokine storm, after which targeted therapy against the underlying trigger is needed to break the cycle of hyperinflammation. Differentiating primary and secondary HLH is paramount, as a hematopoietic stem cell transplant is the only curative option for primary HLH. Often, identifying the underlying cause of hyperinflammation poses a diagnostic dilemma. This is especially true in the case of EBV or HIV infections, which by themselves are triggers for HLH, and can be seen in association with lymphomas. 
